# A Herbal Pair of *Taraxacum officinale* F.H.Wigg. and *Lonicera japonica* Thunb. Ameliorates Obesity and Modulates AMPK Signaling

**DOI:** 10.1002/fsn3.71774

**Published:** 2026-04-15

**Authors:** Seong Chul Jin, You Yeon Choi, Batsukh Sosoburam, Hee Kyung Baek, Min Woo Song, Seungyob Yi, Ji Eun Park, Woong Mo Yang

**Affiliations:** ^1^ Department of Convergence Korean Medical Science, College of Korean Medicine Kyung Hee University Seoul South Korea; ^2^ Korean Medicine Digital Convergence Center (KMDC) Kyung Hee University Seoul South Korea; ^3^ KHU‐KIST Department of Converging Science and Technology, College of Korean Medicine, Graduate School Kyung Hee University Seoul South Korea

**Keywords:** AMPK pathway, fat accumulation, herbal medicine, network pharmacology, obesity

## Abstract

Obesity is a complex metabolic disorder characterized by excessive fat accumulation and associated comorbidities. This study evaluated LIPO‐700, a standardized herbal formulation combining *Lonicera japonica* Thunb. and *Taraxacum officinale* F.H.Wigg., for its anti‐obesity effects and underlying mechanisms. Network pharmacology and KEGG enrichment analyses identified AMPK signaling, adipocytokine signaling, and regulation of lipolysis among the top enriched pathways associated with the predicted targets of LIPO‐700. In vitro, LIPO‐700 significantly reduced lipid accumulation in differentiated 3T3‐L1 adipocytes (up to 19.11% at 100 μg/mL, *p* < 0.001) and free fatty acid‐induced HepG2 hepatocytes (24.70%, *p* < 0.001), accompanied by increased phosphorylation of AMPK (3.49‐fold, *p* < 0.001), restoration of adipose triglyceride lipase (ATGL) and hormone‐sensitive lipase (HSL), and suppression of lipogenic and gluconeogenic markers including Sterol Regulatory Element‐Binding Protein‐1c (SREBP‐1c), Fatty Acid Synthase, Phosphoenolpyruvate Carboxykinase, and Glucose‐6‐Phosphatase. In vivo, oral administration of LIPO‐700 to high‐fat diet‐induced obese mice reduced body weight, fat mass, and adipocyte size in a dose‐dependent manner without hepatotoxicity or nephrotoxicity. Western blot and gene expression analyses of epididymal white adipose tissue showed increased AMPK phosphorylation, together with downregulation of leptin and SREBP‐1c, and upregulation of Lipoprotein Lipase, ATGL, and HSL. These findings demonstrate that LIPO‐700 exerts multi‐target anti‐obesity effects through coordinated regulation of lipid metabolism, supporting its potential as a safe herbal intervention for obesity management.

## Graphical abstract

LIPO‐700, a standardized herbal formulation combining *Taraxacum officinale* F.H.Wigg. and *Lonicera japonica* Thunb., was evaluated for its anti‐obesity potential using an integrative approach. Network pharmacology and KEGG enrichment analyses identified AMPK signaling, adipocytokine signaling, and regulation of lipolysis as key pathways associated with the predicted targets of LIPO‐700. Experimental validation in differentiated 3T3‐L1 adipocytes and free fatty acid (FFA)‐induced HepG2 hepatocytes demonstrated significant reductions in lipid accumulation, accompanied by increased AMPK phosphorylation and suppression of lipogenic and gluconeogenic markers (SREBP‐1c, FAS, PEPCK, G6Pase) alongside upregulation of lipolytic enzymes (ATGL, HSL). In a high‐fat diet (HFD)‐induced obesity mouse model, oral administration of LIPO‐700 significantly reduced body weight, fat mass, and adipocyte size in a dose‐dependent manner, with molecular analyses of epididymal white adipose tissue confirming coordinated modulation of AMPK‐associated metabolic programs. These findings support the potential of LIPO‐700 as a safe multi‐target herbal intervention for obesity management.

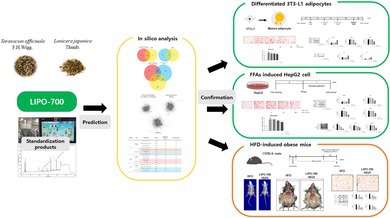

**Abbreviations**
ALT: alanine aminotransferase
AMPK: adenosine monophosphate‐activated protein‐activated protein kinase
AST: aspartate aminotransferase
ATGL: adipose triglyceride lipase
BUN: blood urea nitrogen
DXA: dual X‐ray absorptiometry
eWAT: epididymal white adipose tissue
FAS: fatty acid synthase
FFA: free fatty acids
G6Pase: glucose‐6‐phosphatase
HFD: high‐fat diet
HSL: hormone‐sensitive lipase
LPL: lipoprotein lipase
PEPCK: phosphoenolpyruvate carboxykinase
PPI: protein–protein interaction
SREBP‐1c: sterol regulatory element‐binding protein 1c

## 1 Introduction

Obesity, characterized by excessive accumulation of body fat, poses significant health challenges globally. It is associated with various comorbidities, including hyperlipidemia, type 2 diabetes, atherosclerosis, hypertension, and cardiovascular diseases (Powell‐Wiley et al. 2021). The pathophysiology of obesity involves both adipocyte hypertrophy (enlargement) and hyperplasia (increase in number), which dysregulate lipid metabolism and energy homeostasis (Auger and Kajimura 2023). Adipose tissue dysfunction results in chronic inflammation, insulin resistance, and metabolic abnormalities, necessitating novel therapeutic interventions targeting adipocyte regulation and lipid metabolism (Longo et al. 2019).

Conventional pharmacological treatments for obesity, including orlistat, liraglutide, and phentermine, exert their effects through different mechanisms, such as reducing fat absorption, appetite suppression, or increasing energy expenditure (Pilitsi et al. 2019). However, these drugs often present significant side effects, including gastrointestinal discomfort, cardiovascular complications, and dependence risks (Chakhtoura et al. 2023). These limitations highlight the urgent need for safer and more effective alternatives, particularly those with multi‐target capabilities that address complex pathophysiology. In this context, traditional herbal medicines, which have been used for centuries in East Asia, offer promising candidates for obesity management due to their multi‐target effects and relatively low toxicity.

In traditional Chinese medicine (TCM), obesity is often classified under “dampness‐heat accumulation syndrome” or “phlegm‐dampness retention,” conditions associated with metabolic dysfunction, systemic inflammation, and lipid accumulation (Chen et al. 2019). Herbs with anti‐inflammatory and anti‐oxidative activities, as well as metabolic regulatory effects, have been widely used in traditional practice to manage symptom patterns associated with obesity and metabolic imbalance. Among them, bioactive compounds from herbal medicines have been extensively studied for their potential to modulate lipid metabolism and inflammatory pathways, offering an alternative approach to obesity management.

*Lonicera japonica* F.H.Wigg. ( *L. japonica* ) and *Taraxacum officinale* Thunb. ( *T. officinale* ) are two medicinal plants with established anti‐inflammatory, lipid‐lowering, and metabolic regulatory properties (Hsu et al. 2016). According to ethnopharmacological studies, *Lonicera japonica* has been traditionally used to manage metabolic disorders, including obesity and dyslipidemia, and contemporary pharmacological evidence has described its anti‐inflammatory, anti‐oxidative, and metabolic regulatory activities (de Freitas Junior and de Almeida Jr. 2017). Similarly, *T. officinale* has been reported to aid in reducing fat accumulation and improving liver function, supporting its traditional use in managing metabolic imbalances. Previous studies have shown that *L. japonica* exhibits anti‐obesity potential through its modulation of inflammatory cytokines and lipid metabolism (Hsu et al. 2016), while *T. officinale* demonstrates hepatoprotective and lipolytic activities, supporting its role in metabolic health (Pfingstgraf et al. 2021). Despite their individual pharmacological effects, their combined therapeutic potential in obesity remains unexplored. Given the complementary effects of these two herbs in regulating metabolic pathways, their synergistic potential warrants further investigation. Therefore, we hypothesize that LIPO‐700, a standardized formulation combining *L. japonica* and *T. officinale* , could exert synergistic anti‐obesity effects by modulating key metabolic pathways related to lipid metabolism and adipogenesis.

Recent advancements in network pharmacology have facilitated the systematic investigation of multi‐component herbal formulations by integrating computational predictions with experimental validation. Network pharmacology is particularly useful in herbal medicine research, as it allows the identification of active compounds, prediction of target interactions, and pathway enrichment analysis, providing mechanistic insights into the multi‐target effects of complex botanical formulations (Pujol et al. 2010). By leveraging this approach, it is possible to construct a mechanistic framework for understanding how LIPO‐700 regulates obesity‐related pathways, particularly those involving AMP‐activated protein kinase (AMPK), adipocytokine signaling, and lipolysis.

This study aims to investigate the anti‐obesity potential of LIPO‐700 using an integrative approach combining network pharmacology, in vitro, and in vivo models. By examining its molecular effects on lipid metabolism and energy regulation pathways, we seek to provide a comprehensive evaluation of its therapeutic potential. This study represents one of the first comprehensive assessments of LIPO‐700, providing critical insights into its multi‐targeted effects on lipid metabolism, adipocyte regulation, and systemic energy homeostasis.

## 2 Material and Methods

### 2.1 Sample Preparation

LIPO‐700 consists of two natural herbs, *T. officinale* and *L. japonica* , purchased from Dong‐Yang Herb in Seoul, Korea. A total of 20 kg of *T. officinale* and 30 kg of *L. japonica* were thoroughly cleaned and dried. The botanical identities of *T. officinale* and *L. japonica* were verified using the World Flora Online database (https://www.worldfloraonline.org). The dried herbs, totaling 50 kg, were extracted with 100 L of distilled water at 70°C for 1 h using a mechanical stirrer. The extraction solution was then filtered through a solid suspension filtering apparatus with a 10 μm filter. The filtered solution was concentrated under reduced pressure to a concentration of 16 Brix, resulting in a weight of 47 kg. The concentrated extract was sterilized by heating at 85°C for 20 min, followed by the addition of 11.25 kg of dextrin, constituting 25% of the total mixture, as an excipient. Finally, the mixture was spray‐dried to obtain the powdered form of LIPO‐700. The spray drying conditions were set with an inlet temperature of 180°C, an outlet temperature of 95°C, and an atomizer speed of 10,800 rpm. The yield of LIPO‐700 was 15%, resulting in the production of 7.5 kg of the final product. The LIPO‐700 was contract‐manufactured by HATI Co. Ltd. in Gangwon‐do, Korea, in a GMP‐certified facility. This standardized preparation method ensures the consistent quality and efficacy of the LIPO‐700 extract for further research and application (voucher number: 232829‐002). To determine the optimal combination ratio, various sample ratios ( *L. japonica* : *T. officinale* 5:0, 4:1, 3:2, 2:3, 1:4, 0:5) were tested for their effects on lipid accumulation in HepG2 cells treated with Free Fatty Acids (FFA). The results showed that the 2:3 ratio exhibited the greatest efficacy in reducing lipid accumulation markers (Figure S2).

### 2.2 High Performance Liquid Chromatography Analysis

To ensure the reproducibility and standardized quality of LIPO‐700 across different batches, a comprehensive chemical profiling was conducted using High Performance Liquid Chromatography (HPLC). Loganic acid and Chicoric acid were selected as the specific quantitative marker compounds for *T. officinale* and *L. japonica* , respectively. By quantifying these marker indicators, we verified that the extract maintained a consistent chemical composition meeting the standardization criteria. The HPLC system used was the Thermo Scientific Vanquish UHPLC with Ultimate 3000 Series UV‐Detectors (ThermoFisher Scientific, CA, USA) and an XSelect HSS T3 analytical column (150 × 4.6 mm, 3.5 μm, Waters, MA, USA). The mobile phase consisted of 0.1% formic acid (Sigma‐Aldrich, St. Louis, MO, USA) in water (A) and 0.1% formic acid in acetonitrile (Sigma‐Aldrich, St. Louis, MO, USA) (B), with a gradient program starting at 5% B, increasing to 35% B over 20 min, and then to 100% B, before returning to 5% B. The column temperature was maintained at 40°C, and the detection wavelength was set at 254 nm with a flow rate of 1 mL/min. Loganic acid and chicoric acid standards were prepared in 50% ethanol and distilled water, respectively, and sample solutions were filtered using a PTFE syringe filter (0.22 μm).

### 2.3 Network Construction

To explore the molecular mechanisms underlying the anti‐obesity effects of LIPO‐700, we constructed a Protein–Protein Interaction (PPI) network using the STRING database (Switzerland) with a confidence score of 0.400. The target genes identified for LIPO‐700 were queried, and the resulting interactions were exported to Cytoscape software version 3.9.1 (Cytoscape Consortium, San Diego, CA, USA) for visualization and analysis. The STRING database (v11.5; SIB Swiss Institute of Bioinformatics, Lausanne, Switzerland) integrates known and predicted protein–protein interactions, including direct and indirect associations, providing a comprehensive overview of potential molecular interactions involved in the biological activity of LIPO‐700.

### 2.4 Functional Enrichment Analysis

Biological processes associated with the targets of the LIPO‐700 network were investigated using Cytoscape (version 3.9.1). The pathways related to the LIPO‐700 network were categorized using the Kyoto Encyclopedia of Genes and Genomes (KEGG; Kyoto University/Kanehisa Laboratories, Uji, Kyoto, Japan) pathway 2021 human and Gene Ontology (GO) Process. Relevant biological pathways associated with obesity were selected and organized, and the number of matched genes within each pathway was counted. This analysis provided insights into the molecular mechanisms through which LIPO‐700 may exert its anti‐obesity effects, focusing on key metabolic pathways such as the AMPK signaling pathway, adipocytokine signaling, and the regulation of lipolysis in adipocytes.

### 2.5 Adipogenic Differentiation of 3T3‐L1 Cells and LIPO‐700 Treatment

Mouse preadipocyte 3T3‐L1 cells (CVCL_0123; Cat# CL‐173, ATCC, Manassas, VA, USA) were cultured in Dulbecco's Modified Eagle Medium (DMEM; Gibco, Thermo Fisher Scientific, Grand Island, NY, USA) supplemented with 10% calf serum (Gibco, Thermo Fisher Scientific, Grand Island, NY, USA) and 1% penicillin–streptomycin (Gibco, Thermo Fisher Scientific, Grand Island, NY, USA) at 37°C in a humidified atmosphere containing 5% CO_2_(Jackson et al. 2023; Kaczmarek et al. 2024). Upon reaching confluence (day 0), cells were induced to differentiate using MDI induction medium containing 0.5 mM 3‐isobutyl‐1‐methylxanthine (IBMX; Sigma‐Aldrich, St. Louis, MO, USA), 1 μM dexamethasone (Sigma‐Aldrich, St. Louis, MO, USA), and 10 μg/mL insulin in DMEM supplemented with 10% FBS for 2 days (day 0–2). The medium was then replaced with DMEM containing 10% FBS and 10 μg/mL insulin and incubated for an additional 2 days (day 2–4). From day 4 to day 6, cells were maintained in DMEM with 10% FBS to allow for terminal differentiation. On day 7, fully differentiated adipocytes were treated with LIPO‐700 at concentrations of 1, 10, and 100 μg/mL in DMEM containing 10% FBS. After 24 h of treatment (on day 8), the cells were harvested.

### 2.6 Lipid Accumulation Model Using HepG2 Cells

Human hepatocellular carcinoma HepG2 cells (CVCL_0027; Cat# HB‐8065, ATCC, Manassas, VA, USA) cells were cultured in Dulbecco's Modified Eagle Medium (DMEM) supplemented with 10% fetal bovine serum (FBS) and 1% penicillin–streptomycin at 37°C in a humidified atmosphere containing 5% CO_2_. To induce lipid accumulation, HepG2 cells were seeded at a density of 2 × 10^5^ cells/well in 6‐well plates and allowed to reach 80% confluence. The cells were then exposed to a mixture of palmitic acid (Sigma‐Aldrich, St. Louis, MO, USA) and oleic acid (Sigma‐Aldrich, St. Louis, MO, USA) (final concentration 1 mM) in serum‐free DMEM for 24 h. Following this, the medium was replaced with fresh DMEM containing 10% FBS, and the cells were incubated for an additional 24 h (Lei et al. 2024; Ramos et al. 2022). Following this, the medium was replaced with fresh DMEM containing 10% FBS, and the cells were incubated for an additional 24 h.

Two experiments were conducted: one to compare the effects of different compositions of LIPO‐700 extracts ( *L. japonica* : *T. officinale* ratios of 5:0, 4:1, 3:2, 2:3, 1:4, and 0:5) and another to assess the efficacy of LIPO‐700 extracts at concentrations of 1, 10, and 100 μg/mL. In the composition comparison experiment, the groups included non‐treated control, FFA‐treated control, and FFA‐treated groups with various LIPO‐700 compositions. In the efficacy experiment, the groups included non‐treated control, FFA‐treated control, and FFA‐treated groups with different concentrations of LIPO‐700. After 24 h of treatment, the cells were subjected to various assays to evaluate the effects on lipid metabolism.

### 2.7 Oil Red O Staining

To assess lipid accumulation, Oil Red O (ORO; Sigma‐Aldrich, St. Louis, MO, USA) staining was performed in both HepG2 and 3T3‐L1 cells following their respective treatments. After incubation, cells were washed twice with phosphate‐buffered saline (PBS; Gibco, Thermo Fisher Scientific, Grand Island, NY, USA) and fixed with 10% formaldehyde (Sigma‐Aldrich, St. Louis, MO, USA) for 1 h at room temperature. Fixed cells were rinsed with 60% isopropanol (Sigma‐Aldrich, St. Louis, MO, USA) and stained with freshly prepared Oil Red O working solution (0.5% Oil Red O in isopropanol, diluted with water at a ratio of 3:2) for 30 min at room temperature. Excess stain was removed by washing with 60% isopropanol, followed by several rinses with distilled water. Lipid droplets were visualized under an Olympus microscope (Olympus Corporation, Tokyo, Japan; model [CKX53]) at 400× magnification. For quantification, the stained lipid was eluted with 100% isopropanol, and the absorbance was measured at 520 nm using a microplate reader.

### 2.8 Reverse‐Transcription Polymerase Chain Reaction (RT‐PCR) Analysis

To evaluate the expression levels of genes involved in lipid metabolism, Reverse Transcription Polymerase Chain Reaction (RT‐PCR) was performed on RNA extracted from HepG2 cells, 3T3‐L1 adipocytes, and epididymal White Adipose Tissue (eWAT) of experimental mice. Total RNA was isolated using TRIzol reagent (Thermo Fisher Scientific, MA, USA). Briefly, cells were lysed with TRIzol, mixed with chloroform (Thermo Fisher Scientific, MA, USA), and centrifuged at 12,000 × g for 15 min at 4°C. The aqueous phase was collected, and RNA was precipitated with isopropanol (Thermo Fisher Scientific, MA, USA), washed with 75% ethanol, and resuspended in deionized water. The purity and concentration of RNA were determined using a NanoDrop spectrophotometer (Thermo Fisher Scientific, USA). Isolated total RNA (1 μg) was reverse transcribed into cDNA using Maxime RT Premix (iNtRON Biotechnology Inc., Sungnam, Korea). The synthesis of cDNA was done under the following conditions: stage 1 at 45°C for 60 s and stage 2 at 95°C for 5 min. The cDNA was then amplified with Maxime PCR premix (iNtRON Biotechnology Inc.) and specific primers for target genes such as ACC1, FAS, SREBP‐1c, PPARγ, and the internal control GAPDH. The primers used for the amplification were designed by Bioneer Corp. (Daejeon, Korea) and are listed in Table 1. The relative changes in target gene expression were analyzed using Davinchi‐chemi (Davinchi‐K, Seoul, Korea).

**TABLE 1:** Primer sequences for RT‐PCR.

Species	Genes	Forward primers (5′‐3′)	Reverse primers (5′‐3′)
Mouse	*LPL*	TCCAAGGAAGCCTTTGAGAA	CCATCCTCAGTCCCAGAAAA
	*LEPTIN*	AACCCTTACTGAACTCAGATTGTTAG	TAAGTCAGTTTAAATGCTTAGGG
	*SREBP‐1c*	GGCACTAAGTGCCCTCAACCT	GCCACATAGATCTCTGCCAGTGT
	*GAPDH*	GGCATGGACTGTGGTCATGA	TTCACCACCATGGAGAAGGC
Human	*LPL*	GTCCGTGGCTACCTGTCATT	TGGATCGAGGCCAGTAATTC
	*LEPTIN*	GTGCGGATTCTTGTGGCTTT	GGAATGAAGTCCAAACCGGTG
	*SREBP‐1c*	GGAGGGGTAGGGCCAACGGCCT	CATGTCTTCGAAAGTGCAATCC
	*GAPDH*	CCATCACCATCTTCCAGGAG	CCTGCTTCACCACCTTCTTG

### 2.9 Western Blot Analysis

To evaluate the expression levels of proteins involved in lipid metabolism, Western blot analysis was performed on proteins extracted from HepG2 cells, differentiated 3T3‐L1 adipocytes and eWAT of experimental mice. For HepG2 and 3T3‐L1 cells, total protein was extracted using RIPA buffer (Sigma‐Aldrich, St. Louis, MO, USA) supplemented with protease inhibitor cocktails (Roche, Mannheim, Germany). eWAT was homogenized in tissue protein extraction buffer (Thermo Scientific, Rockford, USA) under the same conditions. The protein concentrations were determined using the Bradford assay solution (Bio‐Rad, CA, USA). A total of 10 μg protein samples were separated by SDS‐polyacrylamide gel electrophoresis (SDS‐PAGE) using appropriate gel percentages (7.5%–12.5%) according to the molecular weights of the target proteins. The polyvinylidene difluoride (PVDF) membranes (0.45 μm; Millipore, Bedford, MA, USA) were blocked with 5% bovine serum albumin (BSA) and incubated overnight at 4°C with primary antibodies against GAPDH, SREBP‐1c, p‐AMPK, PEPCK, G6Pase, ATGL, and HSL (Santa Cruz Biotechnology Inc., Dallas, TX, USA; catalog numbers, RRIDs, host species, and working dilutions are provided in Table S3). After washing, the membranes were incubated for 1 h at room temperature with horseradish peroxidase–conjugated secondary antibodies (Santa Cruz Biotechnology Inc., Dallas, TX, USA; Table S3). The protein bands were then detected using chemiluminescence reagents (AbClon, Seoul, Korea) and visualized with a Chemi‐Doc imaging system (Davinch‐K, Seoul, Korea).

### 2.10 Experimental Animals and Diet

Five‐week‐old male C57BL/6 mice with a body weight of 19–21 g were purchased from RaonBio Inc. (Yongin, Korea). All mice were housed under a 12‐h light/12‐h dark cycle at 22°C ± 2°C and a humidity of 50% ± 5%. All experiments were performed according to the guidelines of the Guide for the Care and Use of Laboratory Animals of the National Institutes of Health and approved by the Committee on Care and Use of Laboratory Animals of Kyung Hee University (KHUASP (SE)‐19‐038). The experimental group had ad libitum access to food and was used after a 7‐day adaptation period. Male mice were randomly assigned to six groups (*n* = 8 per group): normal diet‐fed mice (NOR), high‐fat diet‐fed mice (HFD), HFD‐fed and 10 mg/kg orlistat (Sigma‐Aldrich, St. Louis, MO, USA)‐treated mice (ORL), HFD‐fed and 1.54 mg/kg LIPO‐700‐treated mice (LIPO‐700 LOW), HFD‐fed and 15.4 mg/kg LIPO‐700‐treated mice (LIPO‐700 MID), and HFD‐fed and 154 mg/kg LIPO‐700‐treated mice (LIPO‐700 HIGH). The HFD (Research Diets, D12492) containing 60 kcal% fat was administered for 8 weeks to induce obesity in the mice. LIPO‐700 and orlistat samples were administered 5 times a week for 4 weeks. The human intake doses of LIPO‐700 were converted to mice doses based on the human equivalent dose (HED) established by the guidelines of the FDA. One hundred microliters of corn oil was administered to the HFD group. Orlistat 10 mg/kg and LIPO‐700 at 1.54 mg/kg, 15.4 mg/kg, and 154 mg/kg were diluted or mixed with corn oil, with a final volume of 100 μL for each dose (Figure 4A). At the end of the experimental period, all mice were euthanized using ether and cervical dislocation. Blood samples were collected from the heart under general anesthesia. eWAT was collected and immediately stored at −80°C for further analysis. Body weight and food intake were recorded weekly to monitor the effects of the experimental diets.

### 2.11 Blood Biochemistry Analysis

To assess the potential toxicity of the LIPO‐700 extract, blood samples were obtained via cardiac puncture. The biochemical assessments included measuring levels of blood urea nitrogen (BUN) and creatinine. These measurements were conducted by Dooyeol Biotech Corp. (Seoul, Republic of Korea). Instead of a generic description, the assays were performed using automated enzymatic colorimetric methods. Briefly, BUN was determined using the urease‐glutamate dehydrogenase (GLDH) method, and creatinine was quantified based on the Jaffe reaction (or enzymatic method). The absorbance for each parameter was measured using a standard clinical chemistry analyzer to ensure precision and accuracy.

### 2.12 Dual X‐Ray Absorptiometry Analysis

Dual X‐ray absorptiometry (DXA; medikors, seongnam‐si, Republic of korea) was utilized to evaluate body fat composition in obese mice. This technique involves scanning the animals twice: once with low‐energy X‐rays and once with high‐energy X‐rays, which allows for differentiation of various tissues based on their density. The DXA system categorizes fat tissue into three density levels: high density (red), medium density (yellow), and low density (blue), corresponding to the fat mass. The total fat mass was quantified and expressed in grams, providing a detailed analysis of body fat distribution in the experimental groups.

### 2.13 Histological Analysis

Histological analysis was performed on eWAT to examine fat morphology. The eWAT samples were fixed in 10% formalin, embedded in paraffin, and sectioned at 5 μm thickness. The sections were then stained with hematoxylin and eosin (H&E) to visualize the tissue structure. Fat cell diameters were measured using an Olympus microscope at 400× magnification. This analysis allowed for the assessment of adipocyte size and provided insights into the effects of LIPO‐700 on fat tissue morphology.

### 2.14 Statistical Analysis

Data were presented as the mean ± standard error of the mean (SEM). Statistical significance among multiple groups was assessed using one‐way analysis of variance (ANOVA), followed by Tukey's post hoc test for multiple comparisons. All statistical analyses and graphical plots were performed using GraphPad Prism 5.0 (GraphPad Software Inc., USA).

## 3 Results

### 3.1 Quantification of Marker Compounds in LIPO‐700

To ensure the standardization and consistent quality of LIPO‐700, loganic acid and chicoric acid were established as reference marker compounds for *T. officinale* and *L. japonica* , respectively. Loganic acid was detected at a retention time of 12 min, and chicoric acid at 19.6 min. The average peak areas for loganic acid and chicoric acid were quantified across three replicates, ensuring rigorous quality control (Table S1). The validated HPLC method demonstrated specificity, linearity, and precision, providing accurate quantification of these compounds in LIPO‐700 (Figure S1).

### 3.2 Biological Network Comparison of LIPO‐700 in Obesity

**FIGURE 1:**
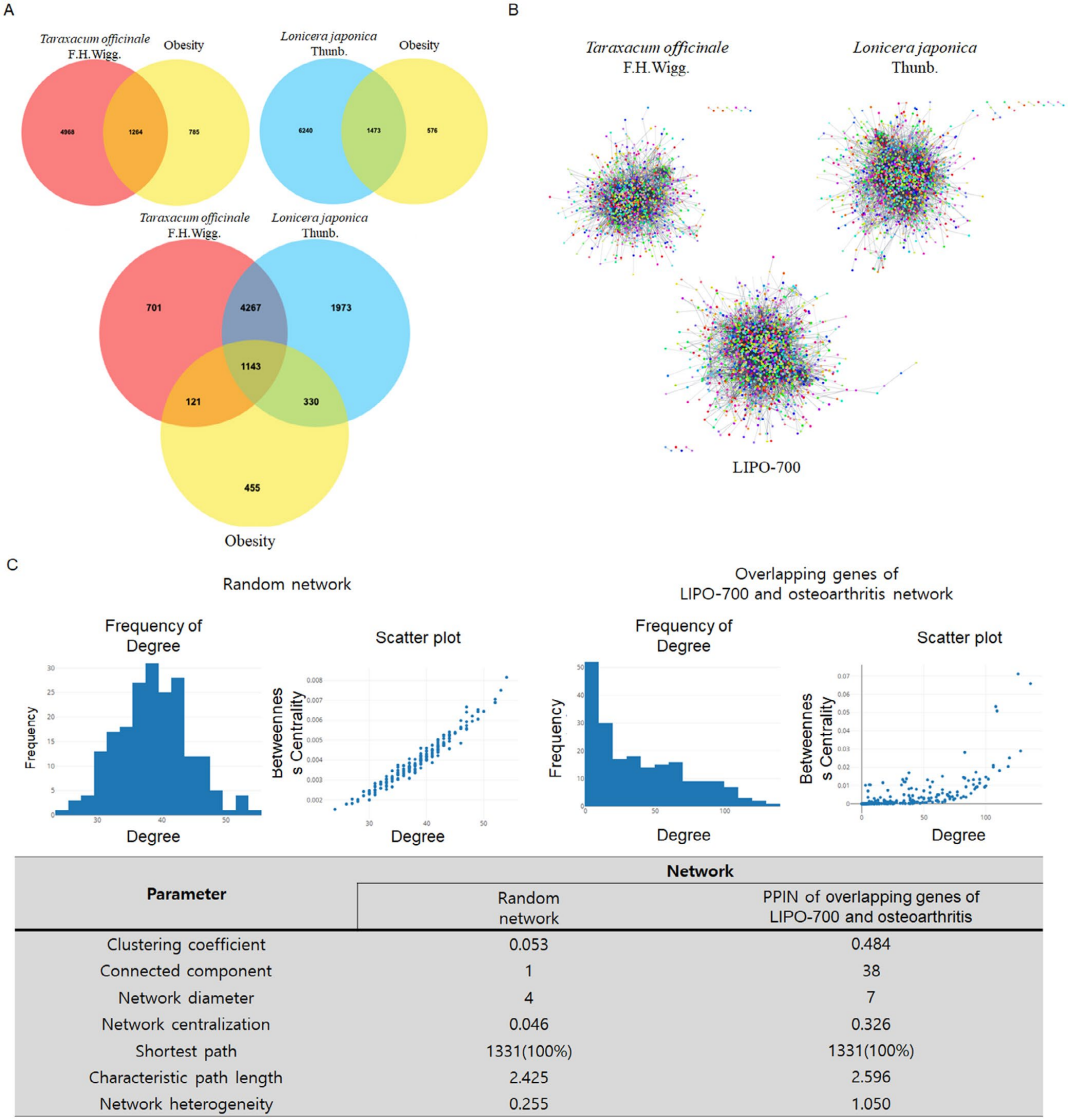
Biological network analysis of LIPO‐700 in obesity. (A) Venn diagrams showing the overlapping target genes among *Taraxacum officinale* F.H.Wigg. ( *T. officinale* ), *Lonicera japonica* Thunb. ( *L. japonica* ), and obesity‐related genes. (B) Protein–protein interaction (PPI) networks of *T. officinale* , *L. japonica* , and LIPO‐700 constructed using Cytoscape software, demonstrating the integration of targets and interactions. (C) Kyoto Encyclopedia of Genes and Genomes (KEGG) pathway maps illustrating the involvement of LIPO‐700 in the AMPK signaling pathway, adipocytokine signaling pathway, and regulation of lipolysis in adipocytes.

By retrieving the Oasis TM‐MC 2.0 database, 135 and 111 related compounds were obtained from *T. officinale* and *L. japonica* , respectively. Altogether, 79 active compounds from *T. officinale* and 45 active compounds from *L. japonica* were screened using oral bioavailability and drug‐likeness (≥ 0.18) filtering criteria (Supporting Information). Through PubChem and STITCH 5.0, we collected the chemical‐gene co‐occurrence data for each compound. By searching Disgenet database with a keyword of “obesity,” we gathered obesity related genes and counted the overlapping genes between the gene set of two herbs and obesity gene set, prioritizing those with relevance scores exceeding 20. The *T. officinale* had 6232 and *L. japonica* had 7713 specific target genes, while obesity had 2447 (Figure 1A). To elucidate synergistic effect, networks were constructed based on active compounds of *T. officinale* and *L. japonica* as well as obesity‐related target genes visualizing interactions among the target genes unveiling unique pathways of LIPO‐700 for treating obesity. PPI networks were constructed through Cytoscape, after inputting targets into the STRING database and removing unlinked targets. Obesity related *T. officinale* consisted of 1262 nodes and 9218 edges, the PPI network of *L. japonica* consisted of 1469 nodes and 11,120 edges, and LIPO‐700 revealed a network of 1590 nodes and 11,898 edges (Figure 1B). Pathways associated with specific diseases in the human disease category were removed to focus on other KEGG categories to provide more informative pathways for the explanation of molecular mechanisms. According to these results, *T. officinale* and *L. japonica* regulated 2 signaling pathways and individually adjusted 2 signaling pathways. The relevant targets of *T. officinale* and *L. japonica* in the AMPK pathways, adipocytokine signaling pathway and regulation of lipolysis in adipocyte are shown in Table 2. The PPIN of overlapping genes between LIPO‐700 and osteoarthritis displayed a markedly higher clustering coefficient (0.484) compared to the random network (0.053), indicating a high degree of interconnectivity among nodes. The network was composed of 38 connected components, with a network diameter of 7 and a characteristic path length of 2.596, slightly longer than that of the random network (2.425). The network heterogeneity value (1.050) was also higher than the random counterpart (0.255), reflecting the presence of hub proteins with disproportionately high connectivity. These parameters suggest that the PPIN exhibited the typical features of a small‐world network, in which most proteins were closely connected through short paths, facilitating efficient information transfer within the system (Figure 1C). Notably, AMPK signaling emerged among the top enriched KEGG pathways in the combined target set.

**TABLE 2:** LIPO‐700 target pathway based on KEGG 2021 human pathway and GO process.

Category	Tool	Description	*p*	Background genes	Common genes
LIPO‐700	GO biological process	Cellular response to lipid	4.63E−41	502	158
		Regulation of lipid metabolic process	6.69E−41	346	131
		Lipid metabolic process	2.66E−38	1210	252
		Regulation of lipid biosynthetic process	1.56E−22	172	68
	KEGG pathways	AMPK signaling pathway	4.60E−17	120	49
		Regulation of lipolysis in adipocytes	7.79E−13	54	28
		Adipocytokine signaling pathway	1.41E−13	68	33
*Taraxacum officinale* Linnear.	GO biological process	Cellular response to lipid	8.41E−40	502	136
		Lipid metabolic process	3.72E−31	918	169
	KEGG Pathways	Regulation of lipolysis in adipocytes	3.75E−10	54	22
		Insulin secretion	7.07E−07	82	21
*Lonicera japonica* Thunb.	GO biological process	Regulation of lipid metabolic process	8.73E−24	155	64
		Regulation of lipid biosynthetic process	3.02E−21	172	63
	KEGG pathways	Adipocytokine signaling pathway	8.48E−14	68	32
		Cholesterol metabolism	9.37E‐10	48	22

### 3.3 Effect of LIPO‐700 on Lipid Metabolism in Differentiated 3T3‐L1 Adipocytes

**FIGURE 2:**
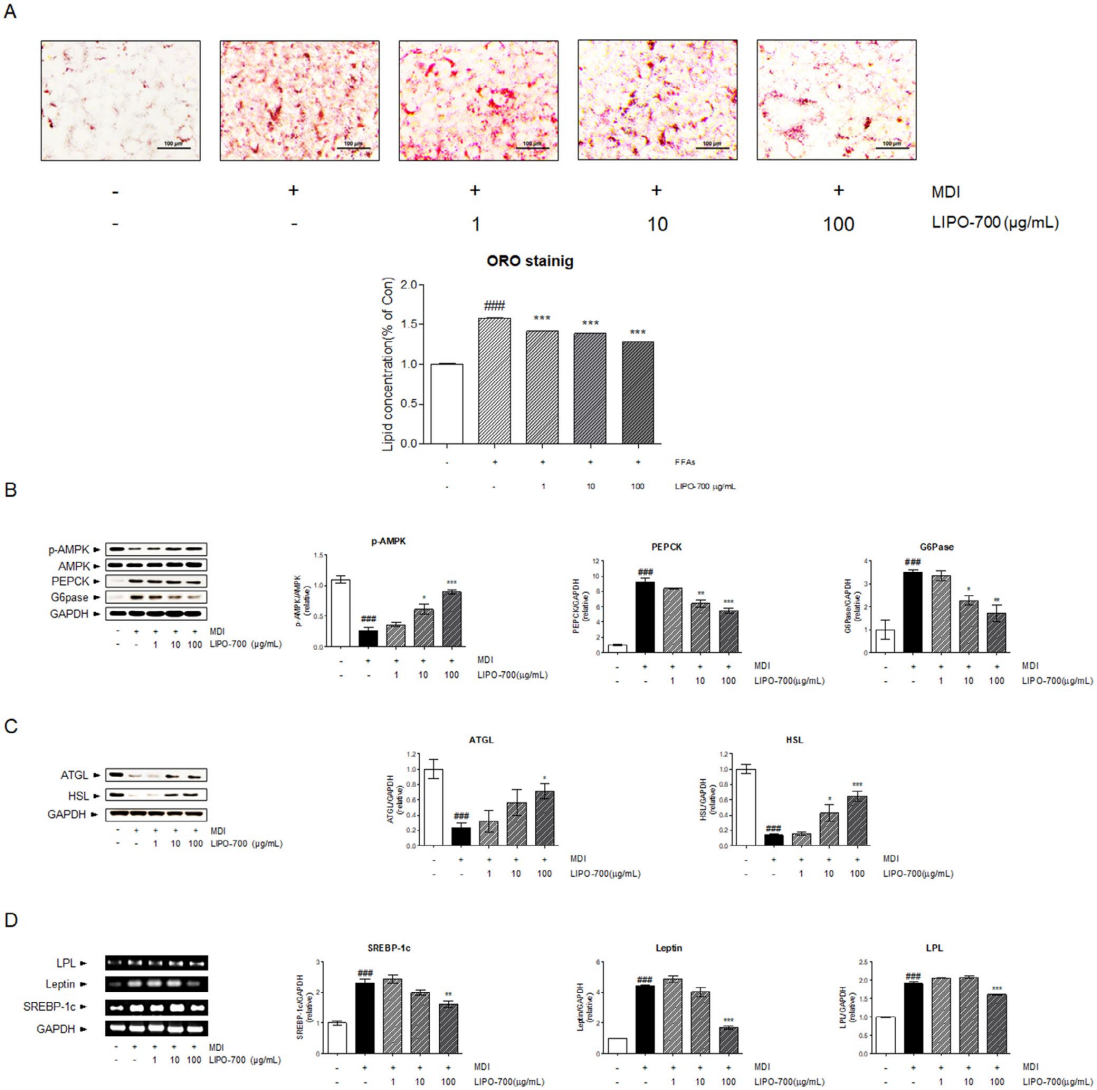
Effect of PL‐700 on lipid accumulation in 3T3‐L1 cells. (A) Oil red O staining of fully differentiated 3T3‐L1 adipocytes treated with LIPO‐700 (1, 10, and 100 μg/mL) for 24 h on day 7 of differentiation. The histogram shows quantification of lipid content expressed as a percentage relative to the MDI‐only group. (B) Western blot analysis of p‐AMPK, AMPK, PEPCK, G6Pase, and GAPDH protein expression in 3T3‐L1 adipocytes after 24‐h treatment with LIPO‐700. (C) Western blot analysis of ATGL and HSL protein expression in 3T3‐L1 adipocytes after 24‐h treatment with LIPO‐700. GAPDH was used as a loading control. (D) RT‐PCR analysis of SREBP‐1c, leptin, and LPL mRNA expression in 3T3‐L1 adipocytes following 24‐h LIPO‐700 treatment. GAPDH was used as an internal control. ###*p* < 0.001, vs. untreated control; *p < 0.05, **p < 0.01, ***p < 0.001, vs. MDI‐treated group.

To further evaluate the anti‐adipogenic effects of LIPO‐700, MDI‐induced 3T3‐L1 adipocytes were treated with 1, 10, and 100 μg/mL of LIPO‐700. Oil Red O staining showed abundant lipid accumulation in the MDI group (###, *p* < 0.001 vs. untreated control). However, treatment with LIPO‐700 significantly reduced this accumulation in a dose‐dependent manner, achieving reductions of 10.25%, 12.9%, and 19.11% at concentrations of 1, 10, and 100 μg/mL, respectively (***, *p* < 0.001 vs. MDI group). Lipid content decreased by 10.25%, 12.9%, and 19.11% at 1, 10, and 100 μg/mL, respectively (***, *p* < 0.001 vs. MDI group) (Figure 2A). Western blot analysis demonstrated that p‐AMPK expression, which was suppressed by MDI induction (###, *p* < 0.001), was significantly increased by LIPO‐700 treatment, with 1.4‐, 2.38‐, and 3.49‐fold increases observed at the respective concentrations (*, *p* < 0.05; ***, *p* < 0.001 vs. MDI group). Conversely, the expression levels of PEPCK and G6Pase, which were upregulated by MDI stimulation (###, *p* < 0.001), were reduced by 8.15%, 29.43%, and 40.04% for PEPCK, and by 4.45%, 35.16%, and 50.96% for G6Pase at 1, 10, and 100 μg/mL of LIPO‐700, respectively (*, *p* < 0.05; **, *p* < 0.01; ***, *p* < 0.001 vs. MDI group) (Figure 2B). Furthermore, the expression of lipolytic enzymes ATGL and HSL, which was significantly suppressed in the MDI group (###, *p* < 0.001), was restored in a concentration‐dependent manner following LIPO‐700 treatment. ATGL levels increased by 1.39‐, 2.45, and 3.09‐fold, while HSL expression rose by 1.09, 3.02‐, and 4.60‐fold, respectively (***, *p* < 0.001 vs. MDI group) (Figure 2C). RT‐PCR analysis showed that SREBP‐1c and leptin expression levels were markedly elevated, whereas LPL expression was suppressed in the MDI group (###, *p* < 0.001 vs. untreated control). RT–PCR analysis showed that SREBP‐1c and leptin mRNA levels were elevated, whereas LPL expression was reduced in the MDI group (###, *p* < 0.001 vs. untreated control). LIPO‐700 decreased SREBP‐1c (5.43%, 13.63%, and 29.64% at 1, 10, and 100 μg/mL, respectively) and leptin (9.35%, 9.53%, and 61.88%), reaching significance at 100 μg/mL (**, *p* < 0.01 vs. MDI group), while LPL expression increased by 15.47% at 100 μg/mL (***, *p* < 0.001 vs. MDI group) (Figure 2D).

### 3.4 Effect of LIPO‐700 on Lipid Accumulation in HepG2 Cells

**FIGURE 3:**
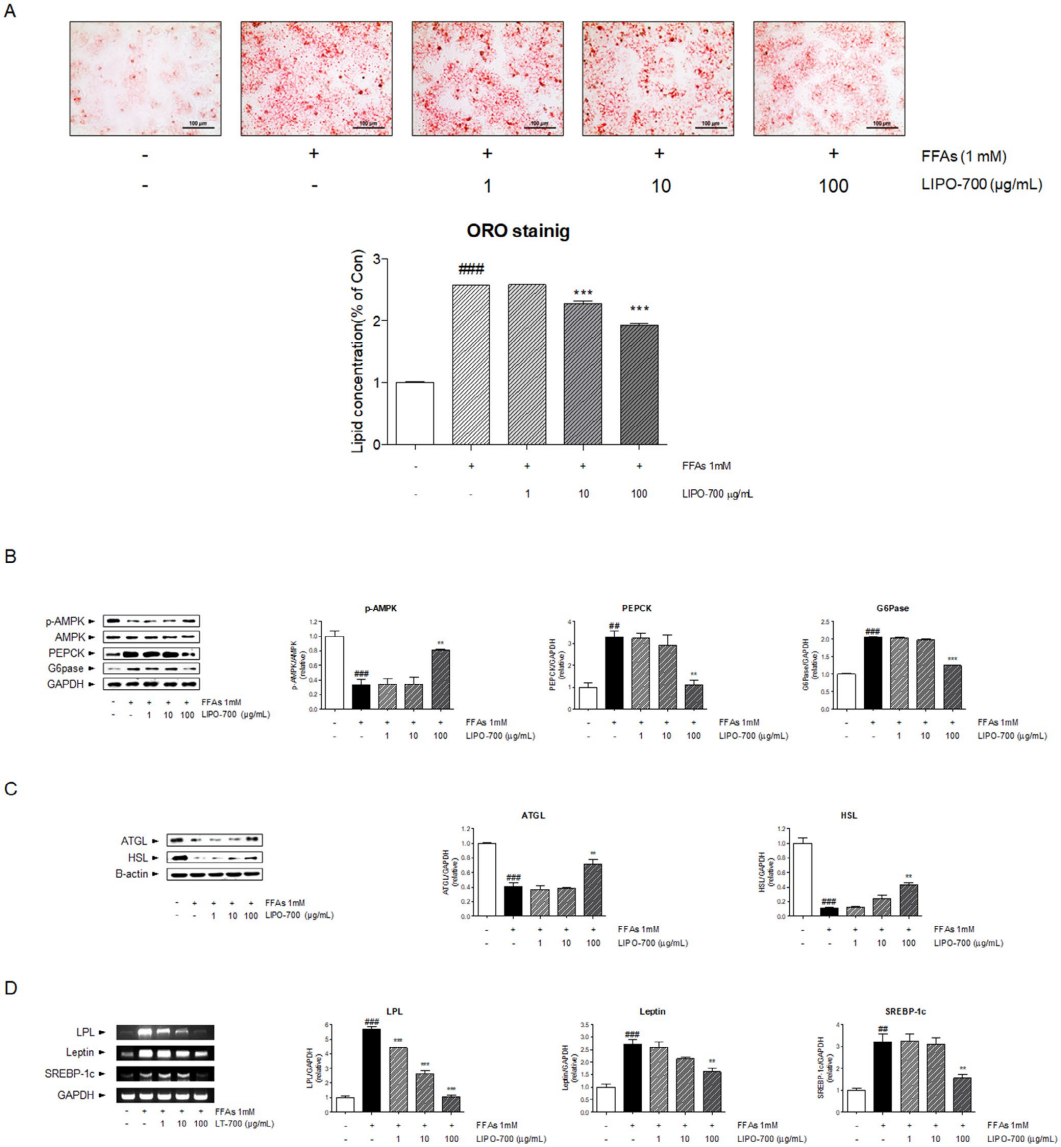
Effect of LIPO‐700 on lipid accumulation in HepG2 cells. (A) Oil red O staining of HepG2 cells treated with free fatty acids (FFA, 1 mM) in the presence or absence of LIPO‐700 (1, 10, and 100 μg/mL) for 24 h. The histogram shows the quantification of lipid content expressed as a percentage of the control group. (B) Western blot analysis of p‐AMPK, AMPK, PEPCK, G6Pase, and GAPDH protein expression in HepG2 cells treated with FFA (1 mM) and LIPO‐700 (1, 10, and 100 μg/mL). (C) Western blot analysis of ATGL and HSL protein expression in HepG2 cells treated with FFA (1 mM) and LIPO‐700 (1, 10, and 100 μg/mL). β‐actin was used as a loading control. (D) RT‐PCR analysis of LPL, leptin, and SREBP‐1c gene expression in HepG2 cells treated with FFA (1 mM) and LIPO‐700 (1, 10, and 100 μg/mL). GAPDH was used as a loading control. GAPDH was used as a loading control. ^###^ *p* < 0.001, vs. control group; **p < 0.01, ***p < 0.001, vs. FFA‐treated group.

Oil Red O staining was performed to assess lipid accumulation in HepG2 cells treated with FFA. The FFA‐treated cells showed significant lipid accumulation, as indicated by the intense staining of lipid droplets. Quantification of staining intensity showed that LIPO‐700 decreased lipid accumulation by 24.70% relative to the FFA–treated group (*, *p* < 0.05) (Figure 3A). Western blot analysis was performed to evaluate the effects of LIPO‐700 on proteins associated with the AMPK pathway in HepG2 cells. In the non‐treated control group, baseline levels of p‐AMPK, ATGL, and HSL were observed, while PEPCK and G6Pase exhibited lower expression. The FFA‐treated group (negative control) showed a significant 66.94% decrease in p‐AMPK, 59.35% decrease in ATGL, and 88.64% decrease in HSL expression (###, *p* < 0.001 vs. untreated control), indicating suppressed lipid catabolism. Concurrently, there was a marked 227% increase in PEPCK and 105.1% increase in G6Pase expression, suggesting enhanced gluconeogenesis compared to the non‐treated group. Treatment with LIPO‐700 at 1, 10, and 100 μg/mL increased p‐AMPK by 1.85%, 4.51%, and 146%, respectively (*, *p* < 0.05; **, *p* < 0.01 vs. FFA group). LIPO‐700 reduced PEPCK levels by 0.12%, 11.53%, and 66.30%, and G6Pase levels by 1.22%, 3.41%, and 39.10% in a dose‐dependent manner (Figure 3B). Conversely, the expression of ATGL changed by 10.18%, 4.70%, and 75.42%, and HSL increased by 114% and 279% following LIPO‐700 treatment (***, *p* < 0.001 vs. FFA group) (Figure 3C). To evaluate the molecular mechanism of LIPO‐700 on lipid metabolism in HepG2 cells, the expression of key lipid‐regulating genes was analyzed by RT‐PCR. The FFA‐treated group exhibited a significant 467.7% increase in LPL, 172.7% increase in leptin, and 219.5% increase in SREBP‐1c compared to the control group (###, *p* < 0.001 vs. untreated control). Treatment with LIPO‐700 reduced LPL by 22.35%, 53.87%, and 81.53%, leptin by 4.66%, 21.78%, and 40.48%, and SREBP‐1c by 1.94%, 2.66%, and 51.02% compared to the FFA‐treated group (*, *p* < 0.05; **, *p* < 0.01; ***, *p* < 0.001 vs. FFA group) (Figure 3D).

### 3.5 LIPO‐700 Reduced Body Weight in HFD‐Induced Obese Mice

**FIGURE 4:**
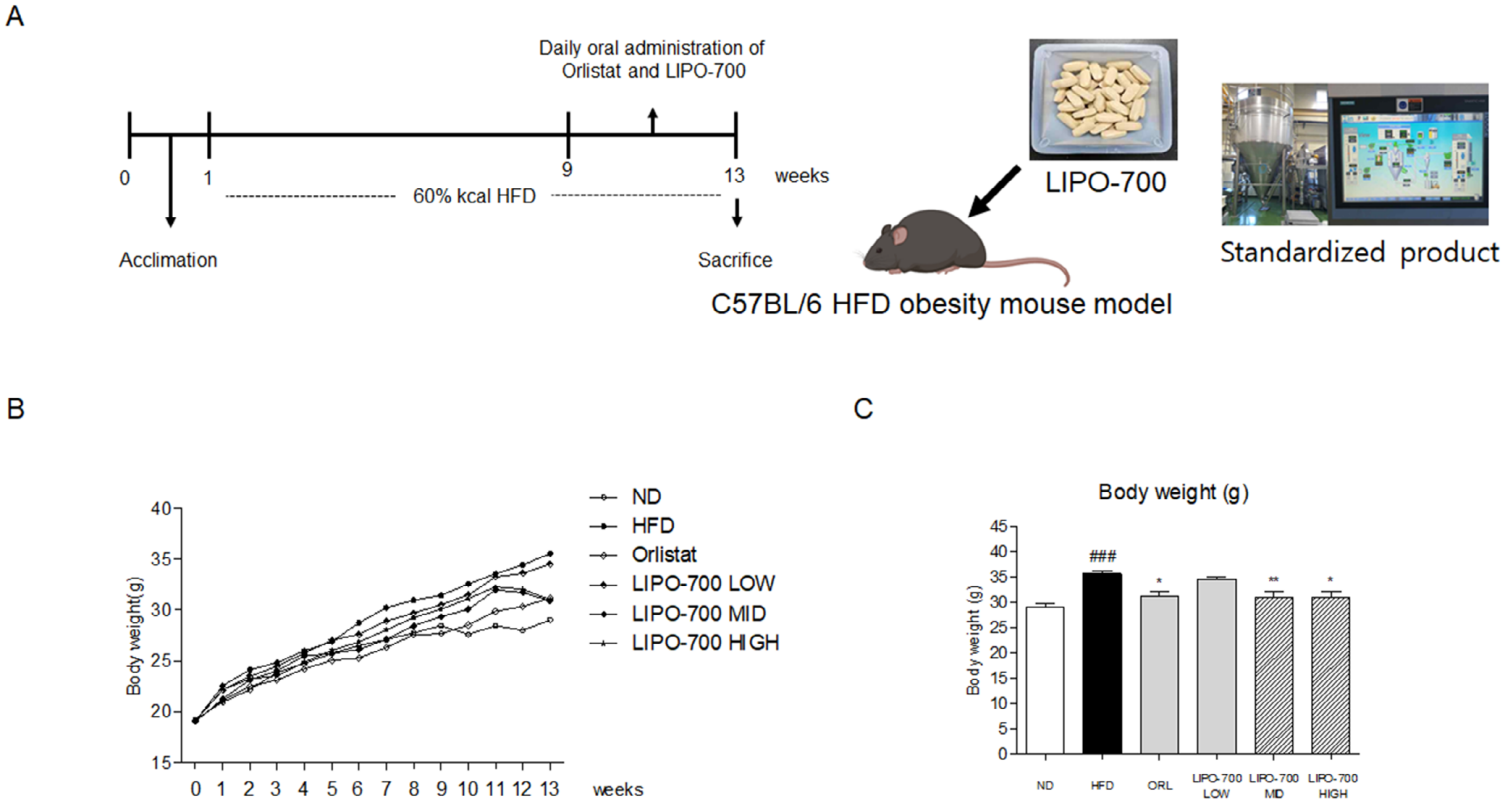
Effect of LIPO‐700 on body weight in HFD‐induced obese mice. (A) Schematic representation of the experimental design. Mice were acclimated for 1 week before being fed a 60% kcal high‐fat diet (HFD) for 13 weeks. Daily oral administration of LIPO‐700 at low, medium, and high doses, as well as Orlistat, was initiated from week 1 until the end of the experiment. (B) Body weight measurements of mice over the 13‐week period, comparing the effects of normal diet (ND), HFD, Orlistat, and different doses of LIPO‐700. (C) Final body weight at the end of the 13‐week treatment period. ^###^ *p* < 0.001, vs. ND group; *p < 0.05, **p < 0.01, vs. HFD group.

The body weight of the HFD group increased significantly compared to the ND group, indicating successful induction of obesity. From weeks 9 to 12, LIPO‐700 was administered at low, mid, and high doses. At the end of the study, the HFD group showed a significant increase in body weight compared to the ND group (Figure 4B). The administration of LIPO‐700 resulted in a dose‐dependent reduction in body weight compared to the HFD group. Specifically, the mid‐ and high‐dose LIPO‐700 groups showed reduced body weight compared with the high‐fat diet group, with a 15% decrease in the high‐dose group (*p < 0.05) (Figure 4C). Orlistat served as a positive control and also significantly reduced body weight, though LIPO‐700 at mid and high doses exhibited comparable efficacy.

### 3.6 LIPO‐700 Reduced Fat Weight in HFD‐Induced Mice

**FIGURE 5:**
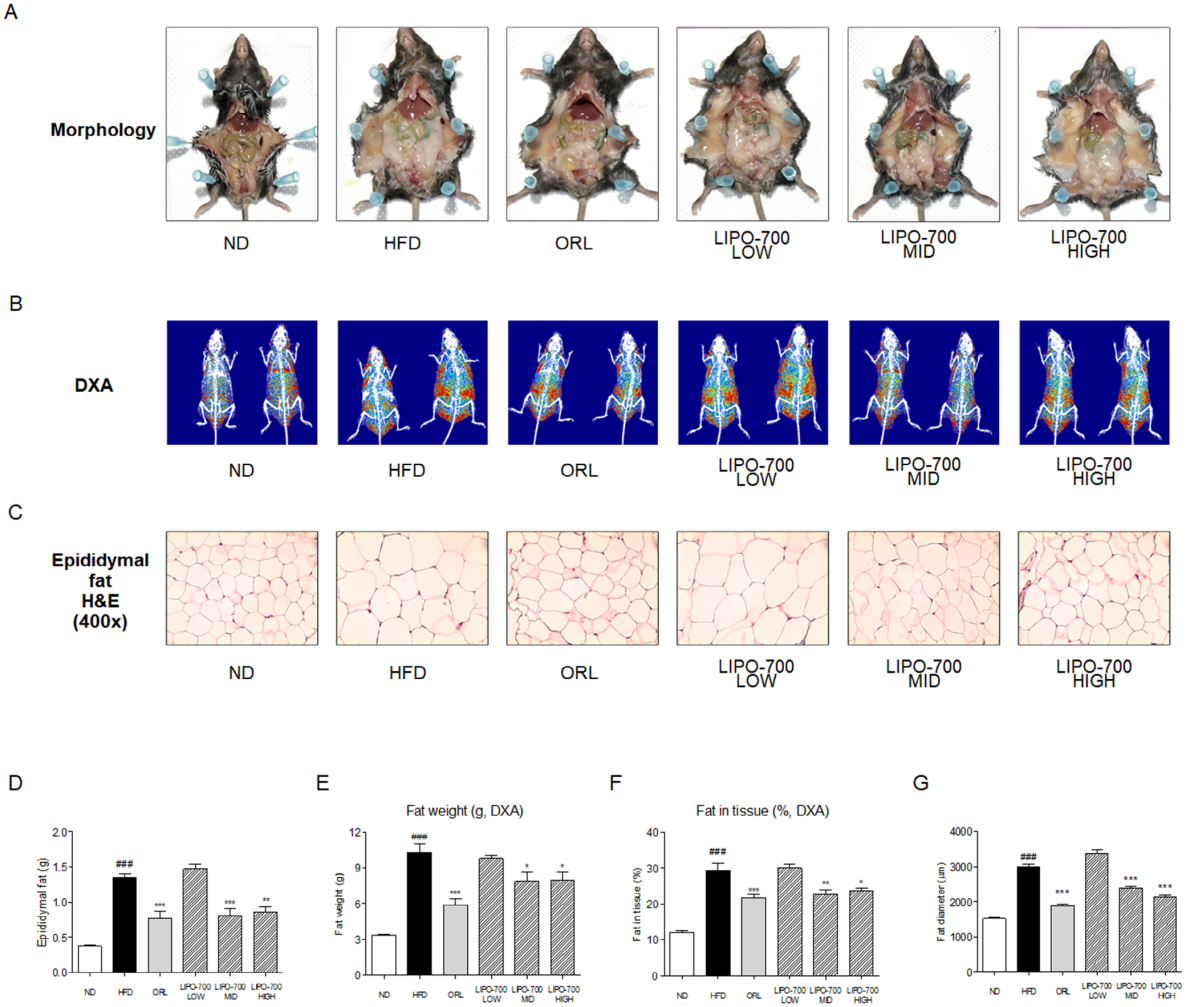
Effect of LIPO‐700 on the weights of fat and epididymal fat in HFD‐induced obese mice. (A) DXA scans showing the distribution of fat mass in mice from different treatment groups, including normal diet (ND), high‐fat diet (HFD), Orlistat (ORL), and LIPO‐700 at low, medium, and high doses. The histograms represent fat weight (g) and fat percentage in tissue (%), as measured by DXA. (B) Representative images of epididymal fat pads from each group. The histogram shows the weight of epididymal fat, demonstrating the effect of LIPO‐700 on fat accumulation. (C) Histological examination of epididymal fat tissue stained with H&E at 400× magnification, showing adipocyte size across different groups. (D) Epididymal fat weight (g) across different treatment groups. (E) Fat weight (g) as measured by DXA across different treatment groups. (F) Fat percentage in tissue (%) as measured by DXA across different treatment groups. (G) Fat diameter (μm) quantifying adipocyte size across different treatment groups, indicating the reduction in adipocyte size with LIPO‐700 treatment. ^###^ *p* < 0.001, vs. ND group; ***p < 0.001, vs. HFD group.

Gross morphological observations of the mice revealed visible differences in body size and abdominal fat accumulation. The HFD group showed a pronounced increase in abdominal girth compared to the ND group, while LIPO‐700 treatment led to a gradual reduction in visible fat mass, particularly in the mid‐ and high‐dose groups (Figure 5A). Consistent with these findings, eWAT weight was measured to evaluate the effects of LIPO‐700 on fat accumulation. The HFD group displayed a substantial increase in eWAT weight compared to the ND group, showing a 3.2‐fold increase. LIPO‐700 treatment significantly reduced eWAT weight in a dose‐dependent manner. The high‐dose LIPO‐700 group demonstrated a 38% reduction in eWAT weight compared to the HFD group (Figure 5D).

DXA was used to assess fat weight and fat percentage in HFD‐induced obese mice. The DXA images use a color‐coded system where red indicates areas of high fat density and blue indicates areas of lower fat density. The HFD group showed a significant increase in red coloration compared to the normal diet (ND) group, indicating a high accumulation of adipose tissue (Figure 5B). Specifically, fat weight and fat percentage increased by approximately 2.5‐fold and 1.8‐fold, respectively, compared to the ND group. Treatment with LIPO‐700 resulted in a dose‐dependent reduction in fat weight and fat percentage. The high‐dose LIPO‐700 group exhibited a notable shift from red to blue areas, reflecting a significant decrease in fat density. Quantitatively, fat weight and fat percentage were reduced by 34% and 27%, respectively, compared to the HFD group (Figure 5E,F). Histological analysis of eWAT weight was conducted to assess changes in adipocyte size. The HFD group showed a marked increase in adipocyte diameter compared to the ND group, reflecting hypertrophic changes associated with obesity (Figure 5C). LIPO‐700 treatment significantly decreased adipocyte diameter in a dose‐dependent fashion. The high‐dose LIPO‐700 group showed a 31% reduction in adipocyte size compared to the HFD group, indicating improved adipocyte morphology and a potential reversal of hypertrophic adipocyte changes (Figure 5G).

### 3.7 LIPO‐700 Reduced Lipid Metabolism Factors in eWAT of HFD‐Induced Obese Mice

**FIGURE 6:**
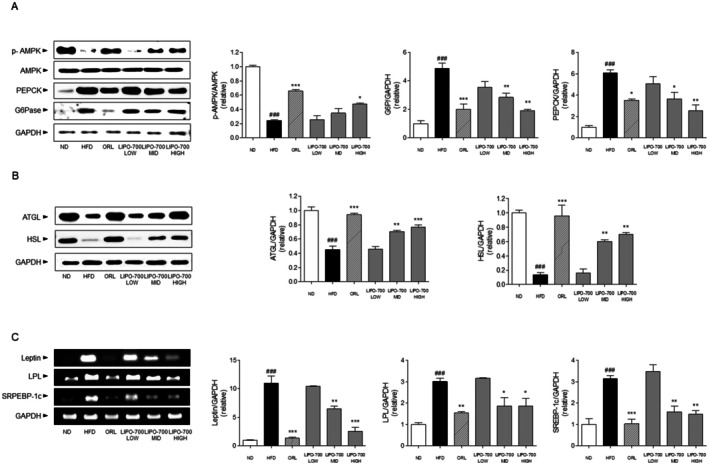
Effect of LIPO‐700 on lipometabolism factors in eWAT of HFD‐induced obese mice. (A) Western blot analysis of p‐AMPK, AMPK, FAS, G6Pase, and PEPCK protein expression in eWAT of mice from different treatment groups, including normal diet (ND), high‐fat diet (HFD), Orlistat (ORL), and LIPO‐700 at low, medium, and high doses. Corresponding densitometric analyses are shown for each protein, normalized to β‐actin. (B) Western blot analysis of ATGL and HSL protein expression in eWAT, with densitometric analyses normalized to β‐actin. (C) RT‐PCR analysis of Leptin, LPL, and SREBP‐1c gene expression in eWAT, with GAPDH used as a loading control. The histograms show relative gene expression levels across different treatment groups. ^###^ *p* < 0.001, vs. ND group; **p < 0.01, ***p < 0.001, vs. HFD group.

Western blot analysis was performed to assess the expression of proteins related to lipid metabolism and gluconeogenesis in eWAT of HFD‐induced obese mice. p‐AMPK expression was significantly reduced in the HFD group (0.34 ± 0.05‐fold) compared to the ND group. LIPO‐700 treatment restored p‐AMPK levels in a dose‐dependent manner, with increases of 5.96%, 42.69%, and 96.30% at low, mid, and high doses, respectively, compared to the HFD group. G6Pase and PEPCK expression was upregulated in the HFD group (2.0 ± 0.18 and 2.3 ± 0.21‐fold, respectively), and decreased following LIPO‐700 treatment. In the high‐dose group, reductions of 46%, 41%, and 38% were observed, respectively, compared to HFD (Figure 6A). ATGL and HSL expression was suppressed in the HFD group (0.42 ± 0.06 and 0.48 ± 0.07‐fold, respectively). LIPO‐700 treatment increased ATGL by 1.8‐, 2.6‐, and 3.2‐fold, and HSL by 1.5‐, 2.1‐, and 2.9‐fold across the dose groups (Figure 6B). The mRNA expression levels of leptin, LPL, and SREBP‐1c in eWAT were evaluated by reverse‐transcription PCR. In the HFD group, leptin and SREBP‐1c expression levels were significantly increased (3.1 ± 0.27‐fold and 3.4 ± 0.23‐fold, respectively), whereas LPL expression was reduced (0.52 ± 0.05‐fold) compared to the ND group. Treatment with LIPO‐700 resulted in a dose‐dependent decrease in leptin and SREBP‐1c expression, with reductions of 32%–51%, while LPL expression was significantly upregulated to 1.4‐, 1.8‐, and 2.2‐fold of the HFD group at low, mid, and high doses, respectively (Figure 6C).

## 4 Discussion

Obesity is a complex metabolic disorder influenced by multiple physiological processes, including adipogenesis, lipid metabolism, and energy regulation. The excessive accumulation of adipose tissue leads to systemic metabolic disturbances, including insulin resistance, dyslipidemia, and chronic low‐grade inflammation (Nasim et al. 2022). While pharmacological interventions such as orlistat have shown efficacy in weight management, concerns regarding long‐term safety, gastrointestinal side effects, and patient adherence highlight the need for alternative therapeutic strategies (Chakhtoura et al. 2023; Pilitsi et al. 2019). In this study, we systematically evaluated the anti‐obesity properties of LIPO‐700 through an integrated network pharmacology and experimental validation approach. Recent advances in network pharmacology and multi‐target drug discovery have provided novel insights into the mechanisms underlying herbal formulations, enabling a more precise evaluation of their therapeutic potential (Leung et al. 2013; Pujol et al. 2010). Our findings suggest that LIPO‐700 exerts anti‐obesity effects via multi‐target regulation of metabolic pathways, accompanied by increased AMPK phosphorylation and coordinated changes in markers related to lipogenesis, gluconeogenesis, and lipolysis, which may collectively contribute to improved metabolic homeostasis. Specifically, AMPK signaling, adipocytokine regulation, and lipogenesis suppression emerged as key enriched pathways in the network pharmacology analysis.

Initial mechanistic insights were obtained through in silico analyses, which identified multiple obesity‐related targets associated with the bioactive compounds present in LIPO‐700. Network pharmacology analysis revealed that this herbal combination interacts with key metabolic regulators, forming an extensive PPI network linked to obesity pathophysiology (Ekins et al. 2007). Specifically, AMPK signaling, adipocytokine regulation, and lipogenesis suppression emerged as key pathways modulated by LIPO‐700. These computational predictions align with previous findings on multi‐component herbal formulations, where active compounds often exert synergistic effects by targeting multiple pathways instead of a single receptor. Further, pathway enrichment analysis suggested that LIPO‐700 has a broader metabolic influence compared to single‐herb treatments, reinforcing the importance of herbal synergy in modulating obesity‐related pathways.

To investigate the cellular mechanisms underlying the metabolic effects of LIPO‐700, 3T3‐L1 cells serve as a standard model for analyzing adipocyte differentiation (Jackson et al. 2023; Kaczmarek et al. 2024) and lipid accumulation, whereas HepG2 cells are widely used to investigate mechanisms of hepatic lipid accumulation and metabolic regulation (Lei et al. 2024; Ramos et al. 2022). In the current study, we employed both models in parallel to validate the regulatory effects of LIPO‐700 on adipose and hepatic lipid metabolism (Chang et al. 2018). In the 3T3‐L1 model, LIPO‐700 treatment significantly reduced lipid accumulation during adipogenic differentiation, as confirmed by Oil Red O staining. This anti‐adipogenic effect was accompanied by increased phosphorylation of AMPK and decreased expression of SREBP‐1c, FAS, PEPCK, and G6Pase, suggesting a shift away from lipogenesis and gluconeogenesis (Rong et al. 2017). These findings are consistent with previous studies reporting AMPK‐mediated inhibition of adipocyte lipid storage during pharmacological or phytochemical interventions. Furthermore, the restoration of ATGL and HSL expression in the presence of LIPO‐700 supports its potential to enhance lipolytic activity, in line with reports demonstrating the role of AMPK in promoting lipid breakdown in adipocytes. The modulation of leptin and LPL expression by LIPO‐700 may also indicate improvements in adipokine signaling and lipid mobilization, which have been associated with healthier adipose tissue function in earlier studies.

Similarly, in HepG2 hepatocytes exposed to FFA to induce steatosis‐like conditions, LIPO‐700 alleviated lipid accumulation and reversed associated molecular alterations. Phosphorylation of AMPK was restored, while FAS, PEPCK, and G6Pase expression was suppressed, in agreement with findings from studies showing the hepatoprotective effects of AMPK activation against lipotoxicity (He et al. 2020). RT‐PCR analysis revealed that LIPO‐700 attenuated the FFA‐induced upregulation of leptin, LPL, and SREBP‐1c, further supporting its role in modulating key transcriptional regulators of hepatic lipid metabolism. These changes suggest that LIPO‐700 may contribute to reducing hepatic lipid burden through mechanisms that have also been proposed in other natural compound‐based interventions targeting the AMPK axis. Taken together, the in vitro data from both adipocyte and hepatocyte models suggest that LIPO‐700 exerts beneficial metabolic effects by modulating lipid metabolic pathways in a manner consistent with AMPK activation and downstream regulation.

To validate the computational findings, in vitro assays were conducted using FFA‐induced HepG2 cells (Yao et al. 2011), a widely accepted model for studying lipid metabolism dysregulation in obesity. Oil Red O staining confirmed that LIPO‐700 significantly reduced intracellular lipid accumulation, while Western blot analysis demonstrated a concomitant increase in AMPK phosphorylation. Given that AMPK activation plays a pivotal role in promoting energy expenditure and lipid oxidation (Foretz et al. 2018; Garcia and Shaw 2017), these findings suggest that LIPO‐700 may help restore metabolic balance under lipid‐overloaded conditions. Additionally, gene expression analysis revealed that LIPO‐700 treatment downregulated adipogenic markers such as SREBP‐1c, LPL, and LEPTIN, which are commonly upregulated in lipid accumulation disorders (Gniuli et al. 2005). Simultaneously, an increase in ATGL and HSL expression was observed, indicating enhanced lipolysis. The suppression of SREBP‐1c, a master regulator of de novo lipogenesis, further suggests that LIPO‐700 mitigates lipid overload by inhibiting triglyceride synthesis while facilitating lipid breakdown (Mihaylova and Shaw 2011). These findings align with previous studies demonstrating that AMPK activation suppresses adipogenic differentiation and promotes mitochondrial fatty acid oxidation, further supporting the role of LIPO‐700 in lipid metabolism regulation.

**FIGURE 7:**
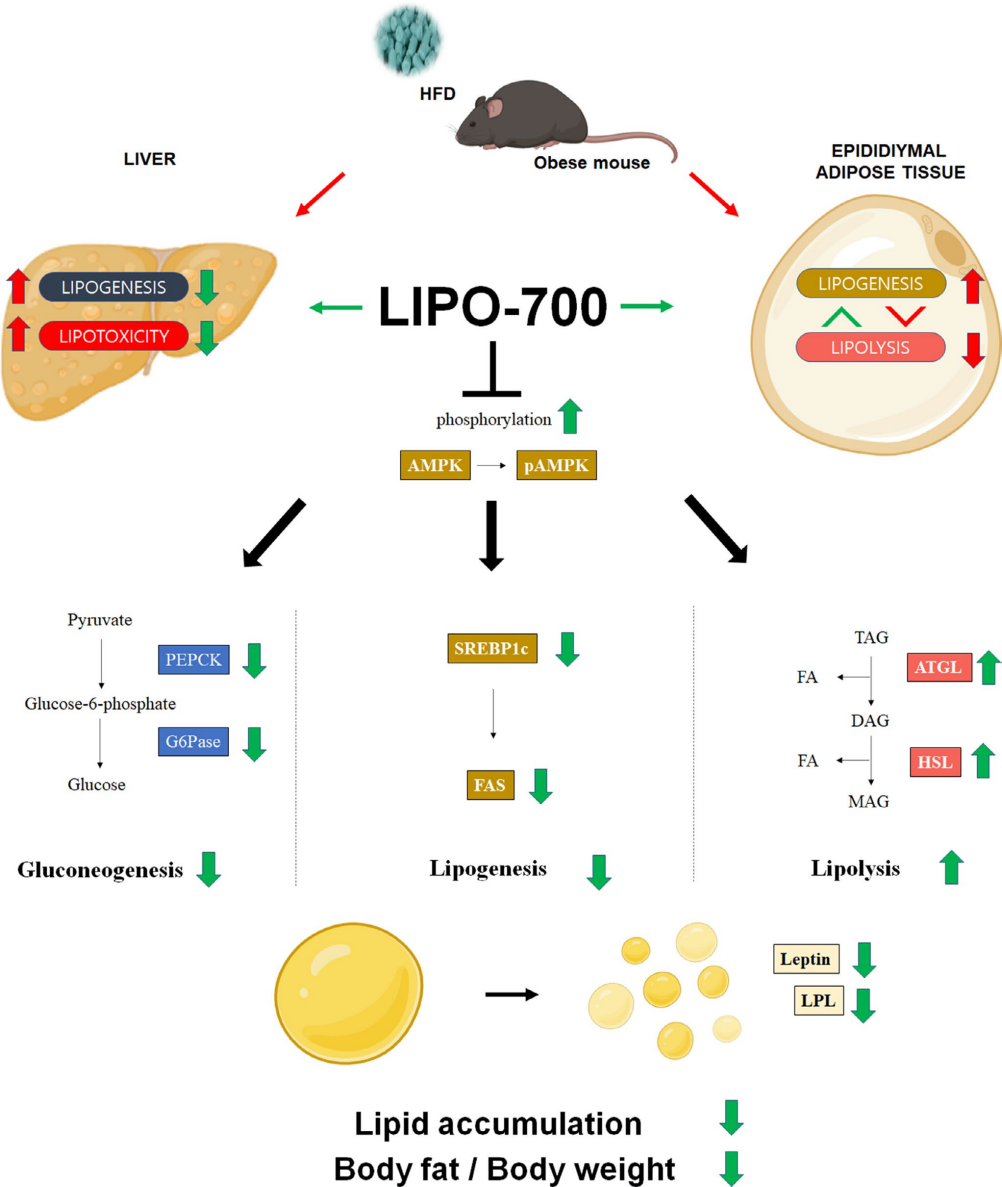
Mechanisms of LIPO‐700 in modulating lipid metabolism. The diagram illustrates the proposed interaction of LIPO‐700 with hepatic and adipose tissue lipid metabolism in a high‐fat diet‐induced obesity model. It shows AMPK activation and phosphorylation, the associated pathways involving lipogenic and gluconeogenic enzymes (SREBP‐1c, FAS, PEPCK, G6Pase), and lipolytic enzymes (ATGL, HSL), as well as their roles in regulating lipid synthesis, breakdown, and distribution between liver and adipose tissue.

To further evaluate the physiological relevance of these findings, in vivo experiments were conducted using an HFD‐induced obesity model, which closely mimics human metabolic syndrome (Muller et al. 2022). Treatment with LIPO‐700 led to a significant, dose‐dependent reduction in body weight and fat mass, with effects comparable to orlistat. Importantly, histological analysis of adipose tissue revealed a marked reduction in adipocyte size, suggesting that LIPO‐700 effectively prevents adipocyte hypertrophy, a key driver of obesity‐related metabolic dysfunctions (Auger and Kajimura 2023). At the molecular level, Western blot analysis of eWAT confirmed increased AMPK phosphorylation, along with downregulation of lipogenic enzymes (FAS, PEPCK, and G6Pase). The ability of LIPO‐700 to enhance fatty acid oxidation while suppressing lipid synthesis further reinforces its role in restoring metabolic homeostasis. A previous study demonstrated that AMPK activation not only enhances lipolysis but also improves mitochondrial function, suggesting that LIPO‐700 effects may extend beyond lipid metabolism and contribute to overall energy homeostasis (Kim et al. 2016). Interestingly, adipokine profiling revealed a reduction in leptin levels, an indicator of improved leptin sensitivity and metabolic flexibility (Obradovic et al. 2021). The concurrent increase in ATGL and HSL expression aligns with findings that AMPK activation enhances lipid mobilization and fatty acid oxidation. Taken together, consistent with our in vitro observations, the coordinated regulation of SREBP‐1c/FAS, PEPCK/G6Pase, and ATGL/HSL demonstrated in vivo further supports the notion that LIPO‐700 induces metabolic remodeling supports the possibility that LIPO‐700 induces metabolic remodeling in association with an AMPK‐centered signaling axis (Figure 7).

Beyond its efficacy in mitigating obesity, the safety of LIPO‐700 was also evaluated. Serum renal markers, including BUN and creatinine, remained within normal physiological ranges (Table S2). In addition, liver histology based on H&E staining showed no apparent signs of hepatocellular injury, such as inflammatory infiltration, necrosis, or structural abnormalities, in the LIPO‐700‐treated groups compared with the HFD‐fed control group (Figure S3). These findings are consistent with the favorable safety context reported for the individual botanical components, including *T. officinale* (Wirngo et al. 2016) and *L. japonica* (Shang et al. 2011), and suggest that herbal formulations may offer a favorable tolerability profile for long‐term obesity management. Nevertheless, pharmacokinetic characterization and well‐designed clinical trials are required to define the optimal dosage, bioavailability, and long‐term safety of LIPO‐700 in humans.

## 5 Conclusions

This study demonstrates that LIPO‐700, a standardized herbal formulation of *T. officinale* and *L. japonica* , ameliorates obesity‐related phenotypes in vitro and in vivo. Network pharmacology and KEGG enrichment analyses highlighted AMPK signaling, along with adipocytokine signaling and regulation of lipolysis, as candidate pathways associated with the predicted targets of LIPO‐700. Experimental validation in differentiated 3T3‐L1 adipocytes, FFA‐treated HepG2 hepatocytes, and HFD‐fed mice showed reduced lipid accumulation and adiposity accompanied by increased AMPK phosphorylation and coordinated regulation of lipid metabolic markers (e.g., SREBP‐1c, FAS, PEPCK, G6Pase, ATGL, and HSL). Collectively, these data suggest that modulation of AMPK‐associated metabolic programs may contribute to the anti‐obesity effects of LIPO‐700.

## Author Contributions

**Seong Chul Jin:** data curation, formal analysis, investigation, methodology, resources, software, validation, visualization, writing – original draft. **You Yeon Choi:** formal analysis, validation, writing – review and editing. **Batsukh Sosoburam:** formal analysis, data curation. **Hee Kyung Baek:** formal analysis, investigation. **Min Woo Song:** data curation, formal analysis. **Seungyob Yi:** writing – review and editing, formal analysis, methodology. **Ji Eun Park:** writing – review and editing, formal analysis. **Woong Mo Yang:** conceptualization, funding acquisition, methodology, project administration, supervision.

## Funding

This research was supported by the Health Functional Food Development Support Project (R&D) funded by the Ministry of SMEs and Startups, Republic of Korea (RS‐2022‐00167188), and by the National Research Foundation of Korea (NRF) grant funded by the Korean government (RS‐2025‐00522776).

## Conflicts of Interest

The authors declare no conflicts of interest.

## Supporting information


**Figure S1:** LIPO‐700 HPLC analysis.
Representative extracted ion chromatograms (EIC) of standard compounds (loganic acid and chicoric acid) in negative ion mode, along with their corresponding MS/MS fragmentation spectra. The lower panels show extracted ion chromatograms of LIPO‐700 samples from three independent lots (LotA1, LotA2, and LotA3), confirming the consistent presence and retention times of loganic acid and chicoric acid across batches.

**Figure S2:** Western blot analysis of p‐AMPK and AMPK in HepG2 cells treated with varying composition ratios of LIPO‐700.HepG2 cells were treated with free fatty acids (FFA, 1 mM) and varying ratios of *Taraxacum officinale* (TO) and *Lonicera japonica* (LJ) extracts at a total concentration of 100 μg/mL. Representative western blot images and densitometric quantification of p‐AMPK and AMPK expression are shown. GAPDH was used as a loading control. ###*p* < 0.001, vs. untreated control; *p < 0.05, **p < 0.01, ***p < 0.001, vs. FFA‐treated group.

**Figure S3:** Histological toxicity evaluation of major organs after treatment.
Representative H&E‐stained sections of liver and kidney tissues at 400× magnification from mice in each treatment group, including normal diet (ND), high‐fat diet (HFD), Orlistat (ORL), and LIPO‐700 at low, medium, and high doses. No apparent histopathological abnormalities were observed in LIPO‐700‐treated groups compared with the HFD control group.

**Data S1:** Compounds of LIPO‐700 in TM‐MC database. Data S2. Compounds and their related genes of LIPO‐700. Data S3. Related genes of LIPO‐700. Data S4. Sorted genes of LIPO‐700 via cytoscape string app with confidence score 0.7. Data S5. Functional enrichment analysis of LIPO‐700.

**Table S1:** Peak area of Loganic acid and Chicoric acid.
**Table S2:** Serum toxicity evaluation of major organs after treatment.
**Table S3:** Antibody information.

## Data Availability

The data that support the findings of this study are available on request from the corresponding author. The data are not publicly available due to privacy or ethical restrictions.
