# Supplementary figures and images for: A Herbal Pair of Taraxacum officinale F.H.Wigg. and Lonicera japonica Thunb. Ameliorates Obesity and Modulates AMPK Signaling
Source: Food Sci Nutr. 2026 Apr 15;14(4):e71774. doi: 10.1002/fsn3.71774 (PMC13082916; doi:10.1002/fsn3.71774)

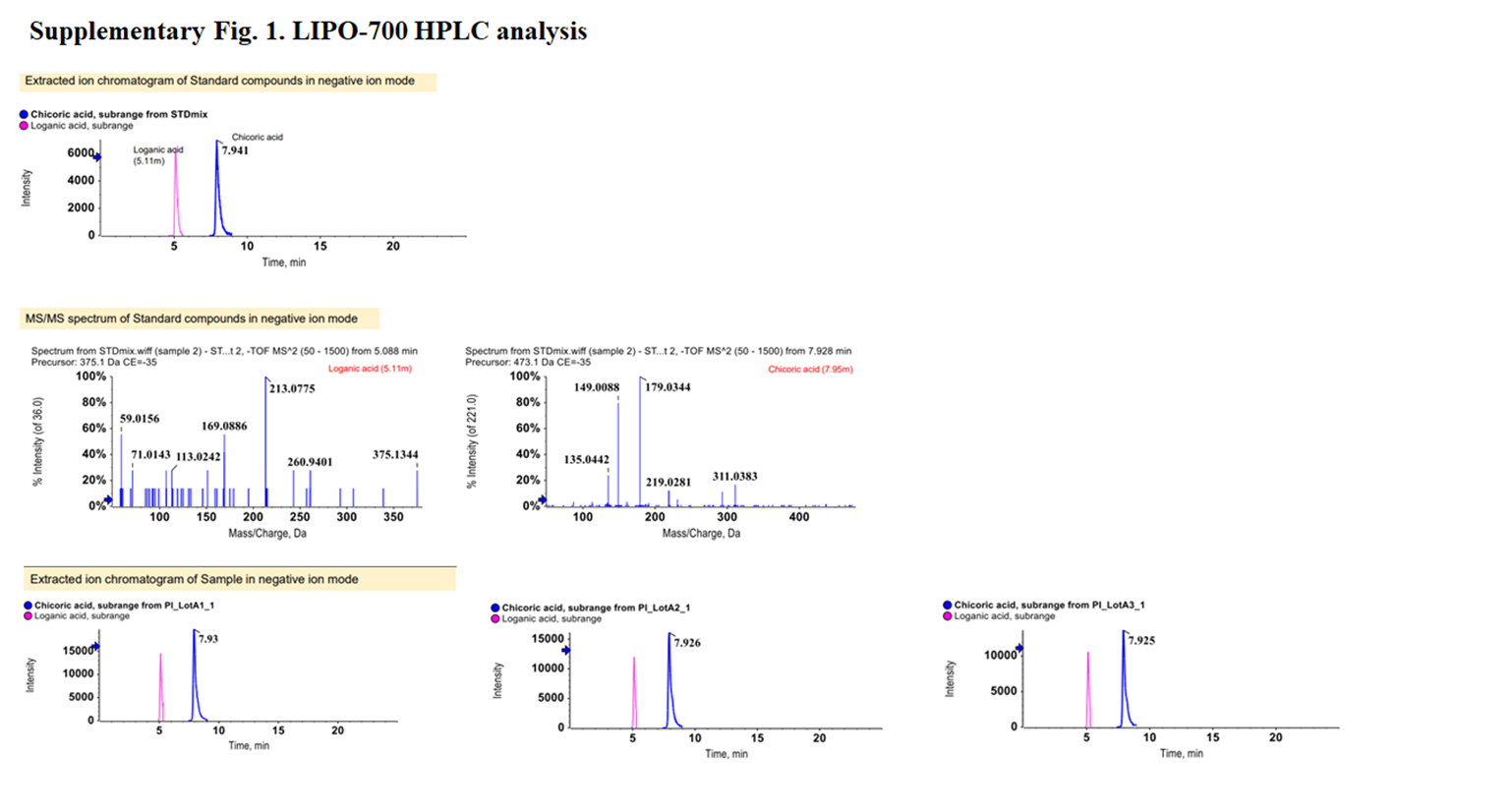

Supplement: Supplementary file 1 — Figure S1: LIPO‐700 HPLC analysis. Representative extracted ion chromatograms (EIC) of standard compounds (loganic acid and chicoric acid) in negative ion mode, along with their corresponding MS/MS fragmentation spectra. The lower panels show extracted ion chromatograms of LIPO‐700 samples from three independent lots (LotA1, LotA2, and LotA3), confirming the consistent presence and retention times of loganic acid and chicoric acid across batches. [file FSN3-14-e71774-s004.tif]

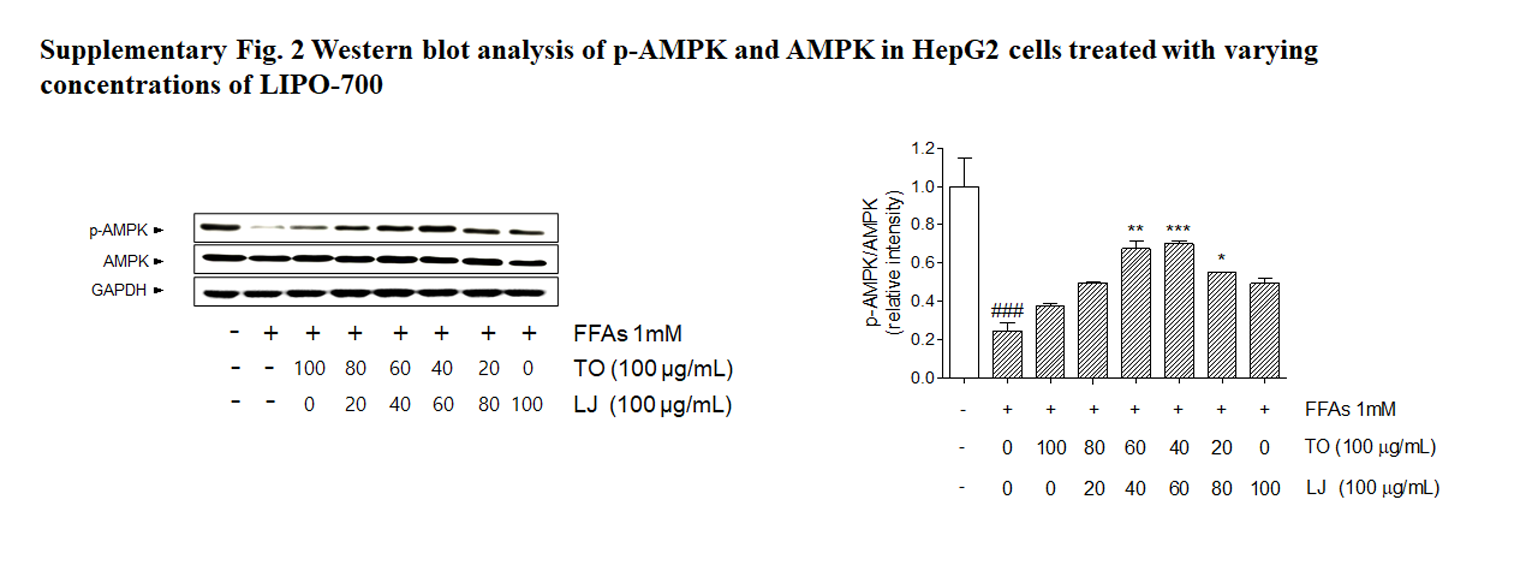

Supplement: Supplementary file 2 — Figure S2: Western blot analysis of p‐AMPK and AMPK in HepG2 cells treated with varying composition ratios of LIPO‐700.HepG2 cells were treated with free fatty acids (FFA, 1 mM) and varying ratios of Taraxacum officinale (TO) and Lonicera japonica (LJ) extracts at a total concentration of 100 μg/mL. Representative western blot images and densitometric quantification of p‐AMPK and AMPK expression are shown. GAPDH was used as a loading control. ###p < 0.001, vs. untreated control; *p < 0.05, **p < 0.01, ***p < 0.001, vs. FFA‐treated group. [file FSN3-14-e71774-s001.tif]

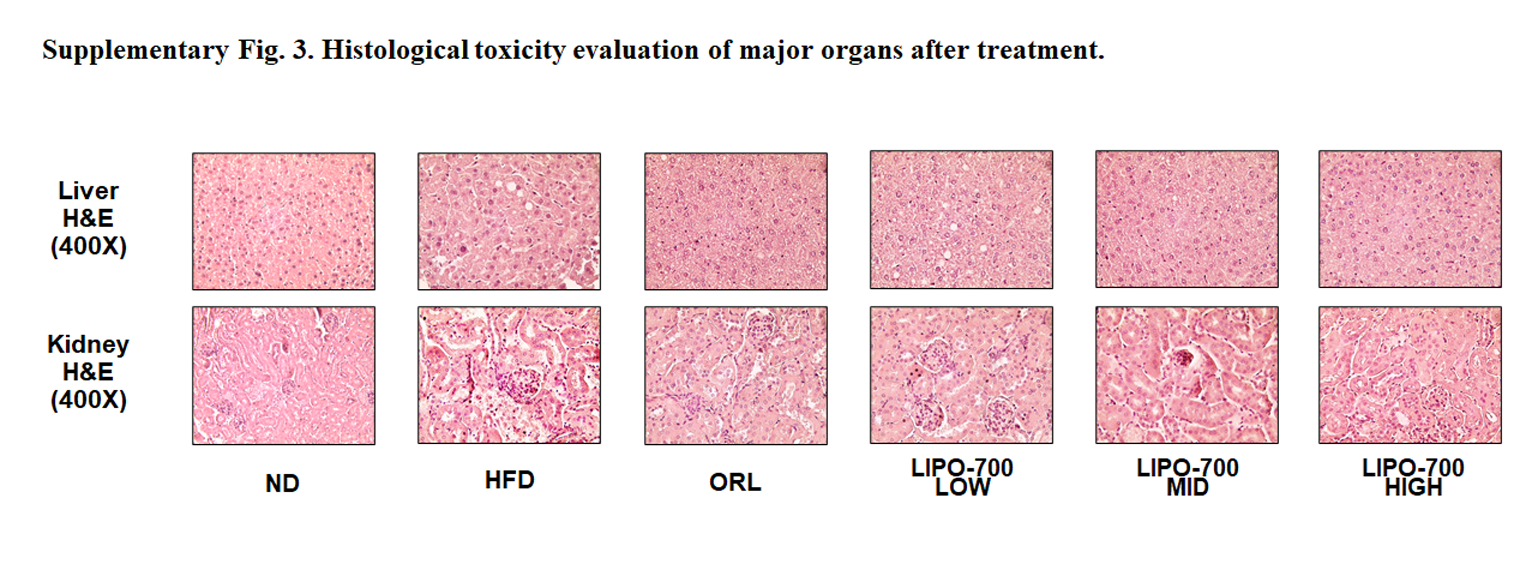

Supplement: Supplementary file 3 — Figure S3: Histological toxicity evaluation of major organs after treatment. Representative H&E‐stained sections of liver and kidney tissues at 400× magnification from mice in each treatment group, including normal diet (ND), high‐fat diet (HFD), Orlistat (ORL), and LIPO‐700 at low, medium, and high doses. No apparent histopathological abnormalities were observed in LIPO‐700‐treated groups compared with the HFD control group. [file FSN3-14-e71774-s002.tif]
