# Supplementary material for: A Herbal Pair of Taraxacum officinale F.H.Wigg. and Lonicera japonica Thunb. Ameliorates Obesity and Modulates AMPK Signaling
Source: Food Sci Nutr. 2026 Apr 15;14(4):e71774. doi: 10.1002/fsn3.71774 (PMC13082916; doi:10.1002/fsn3.71774)
Supplement: Supplementary file 5 — Table S1: Peak area of Loganic acid and Chicoric acid. Table S2: Serum toxicity evaluation of major organs after treatment. Table S3: Antibody information. [file FSN3-14-e71774-s003.docx]

**Supplementary Table. 1. Peak area of Loganic acid and Chicoric acid**

| Sample | | Loganic acid | | | Average | Chicoric acid | | | Average |
| --- | --- | --- | --- | --- | --- | --- | --- | --- | --- |
|  |  | #1 | #2 | #3 |  | #1 | #2 | #3 |  |
| Lot A | LotA1 | 1731 | 1812 | 1882 | 1539 | 4880 | 5222 | 5666 | 4377 |
|  | LotA2 | 1398 | 1506 | 1577 |  | 4180 | 4216 | 4524 |  |
|  | LotA3 | 1248 | 1337 | 1357 |  | 3456 | 3500 | 3746 |  |

**Supplementary Table. 2. Serum toxicity evaluation of major organs after treatment.**

|  | NOR | HFD | ORL | PL-700 LOW | PL-700 MID | PL-700 HIGH |
| --- | --- | --- | --- | --- | --- | --- |
| BUN | 29.37 ± 3.97 | 31.06 ± 3.82 | 29.92 ± 3.46 | 32.38 ± 1.96 | 28.38 ± 4.35 | 32.44 ± 3.13 |
| CREATINE | 0.33 ± 0.04 | 0.42 ± 0.15 | 0.39 ± 0.03 | 0.40 ± 0.04 | 0.38 ± 0.02 | 0.38 ± 0.02 |

**Supplementary Table. 3. Antibody information**

| **Antibody** | | **Dilution** | **Source** | **RRID** | **Cat no.** | **Company** |
| --- | --- | --- | --- | --- | --- | --- |
| Primary | GAPDH | 1:1000 | Mouse | AB_627679 | sc-32233 | Santa-cruz |
|  | PPARγ | 1:1000 | Mouse | AB_628115 | sc-7273 | Santa-cruz |
|  | AMPK alpha 1/2 | 1:1000 | Mouse | AB_1118940 | sc-74461 | Santa-cruz |
|  | p-AMPK alpha | 1:1000 | Rabbit | AB_330330 | #2531 | Cell signaling |
|  | PEPCK | 1:1000 | Mouse | AB_10610383 | sc-271029 | Santa-cruz |
|  | G6Pase | 1:1000 | Rabbit | AB_10903775 | ab93857 | Abcam |
|  | ATGL | 1:1000 | Mouse | AB_10859044 | sc-365278 | Santa-cruz |
|  | HSL | 1:1000 | Rabbit | AB_2135501 | sc-25843 | Santa-cruz |
| Secondary | Goat Anti-Rabbit IgG H&L (HRP) | 1:3000 | Goat | AB_955447 | ab6721 | Abcam |
|  | Goat Anti-Mouse IgG H&L (HRP) | 1:3000 | Goat | AB_955439 | ab6789 | Abcam |
